# Epidemiology and Drug Resistance of Fracture-Related Infection of the Long Bones of the Extremities: A Retrospective Study at the Largest Trauma Center in Southwest China

**DOI:** 10.3389/fmicb.2022.923735

**Published:** 2022-07-12

**Authors:** Zhengdong Zhang, Pan Liu, Wenzhao Wang, Shanxi Wang, Bohua Li, Jun Li, Banyin Yang, Mingxin Li, Qin Li, Hai Yang, Zeyu Huang, Lei Liu

**Affiliations:** ^1^School of Clinical Medicine, Chengdu Medical College, Chengdu, China; ^2^Department of Orthopedics, The First Affiliated Hospital of Chengdu Medical College, Chengdu Medical College, Chengdu, China; ^3^School of Clinical Medicine, Chengdu University of Traditional Chinese Medicine, Chengdu, China; ^4^Department of Orthopedics, West China Hospital, Sichuan University, Chengdu, China; ^5^Department of Orthopedics, Tongji Hospital, Tongji Medical College, Huazhong University of Science and Technology, Wuhan, China

**Keywords:** fracture-related infection, long bone fracture, extremities, risk factors, epidemiology, drug resistance

## Abstract

**Objective:**

To describe the demographic characteristics, risk factors, and bacterial resistance of fracture-related infection (FRI) of the long bones of the extremities.

**Materials and Methods:**

This single-center study retrospectively evaluated patients with FRI of the long bones of the extremities at West China Hospital between January 2012 and December 2017, and analyzed the demographic characteristics, risk factors, distribution of pathogenic bacteria, and bacterial drug resistance.

**Results:**

Among 9,900 patients, 535 patients (5.4%) were diagnosed with FRI. The most common site of FRI was tibiofibular (298, 55.7%), with 424 cases (79.2%) of open fractures, and 282 cases (52.7%) due to traffic injuries. The 41–50 years age group had the highest incidence of FRI with 157 (29.3%) cases. Overall, 546 strains of 52 types of bacteria were detected in FRI patients, with 105 strains of multidrug-resistant (MDR) bacteria. Methicillin-resistant *Staphylococcus aureus* (48, 8.8%) and extended-spectrum-β-lactamase *Escherichia coli* (32, 5.8%) accounted for the largest proportion. Multivariate logistic regression analysis showed that sex (odds ratio [OR] 1.813; 95% confidence interval [CI], 1.071∼3.070; *P* = 0.027) and fracture type (OR 3.128; 95% CI, 1.683∼5.815; *P* < 0.001) were independent risk factors for monomicrobial infection (MI). Female sex (OR 4.190; 95% CI, 1.212∼14.486; *P* = 0.024) was an independent risk factor for polymicrobial infection (PI).

**Conclusion:**

This study clarified the infection rates, changes in the bacterial spectrum, and drug resistance characteristics, and risk factors of FRI of the long bones of the extremities in the largest trauma center in southwest China.

## Introduction

Fracture-related infection (FRI) is a common complication of long bone fractures of the extremities. In severe cases, multiple operations or even amputation may be required to save the patient’s life, causing a major problem for orthopedic surgeons as well as a serious social and economic burden (Metsemakers et al., 2017; Baecker et al., 2020). FRI may have a variety of causes, and previous studies have shown that the risk of infection after a fracture often depends on the sex, severity of the injury, lower extremity fractures, and the overall physical condition of the patient (Kortram et al., 2017; Depypere et al., 2020). At the same time, FRI is more common in developing countries than in developed countries (Walter et al., 2021). Previous research has shown that the incidence of bone infection is approximately 15–30% in complex open fractures and 1% in closed low-energy fractures (Morgenstern et al., 2018a; Puetzler et al., 2019).

Due to the diversity of clinical characteristics, the definition of FRI varies in different countries and regions (Morgenstern et al., 2018b). In 2018, organizations including the European Bone and Joint Infection Society and the Association for the Study of Internal Fixation worked together to develop a consensus on FRI to standardize the clinical reports and improve the quality of published literature (Metsemakers et al., 2018; Govaert et al., 2020). Despite advances in the treatment of FRI, the diagnosis and treatment of FRI are still in the process of continuous exploration due to its diversity and complexity (Foster et al., 2020; Hak, 2020; Hotchen et al., 2020; Metsemakers et al., 2020). Understanding the epidemiology and risk factors for FRI may lead to better prevention and treatment methods in the future. This retrospective study aimed to analyze the fracture types and sites, injury causes, microbiological data, and drug resistance of FRI in the long bones of the extremities. In addition, we also analyzed the risk factors for FRI in order to provide an evidence-based medical basis for the management of FRI of the long bones of extremities.

## Materials and Methods

### Study Design

**FIGURE 1:**
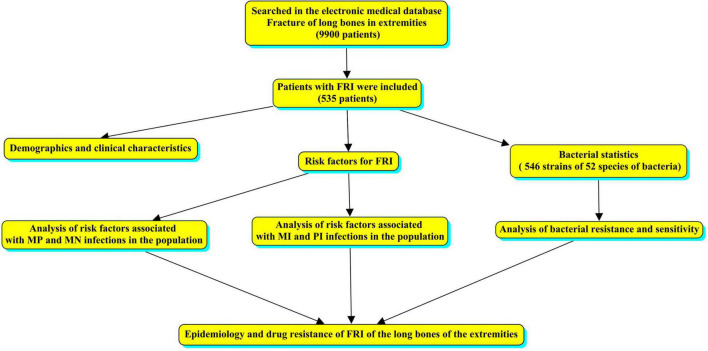
The design of the overall flow chart of this study.

We conducted a single-center retrospective study of patients treated at West China Hospital from January 2012 to December 2017. Cases with a diagnosis of limb fracture were searched in the electronic medical database and sex, age, cause of injury, fracture site, fracture type, comorbidities, bacterial culture results, and bacterial susceptibility testing were analyzed. No changes in the reporting system occurred during the study period; therefore, all cases of long bone fractures of the limbs with or without bone infection were collected. The overall flow chart was made by CmapTools software (Behzadi and Gajdacs, 2021), as shown in Figure 1.

### Clinical Definitions

Based on the latest definition of FRI, the diagnostic criteria of FRI of limbs were clarified as: (A) Fistula, sinus, or wound dehiscence; (B) Pus drainage or exudation was found during the operation; (C) No less than four parts of the deep tissue suspected of being infected were sampled during the operation for obtaining positive bacterial culture; and (D) Histopathological examination revealed the presence of pathogenic microorganisms in the deep tissues during the operation. If one of the above conditions was met, FRI was considered (Metsemakers et al., 2018).

### Microbiological Examination

Detection standards for pathogenic bacteria and drug sensitivity tests: Culture medium and reagent: Vitek-2 Compact (Bio-Merieux, France) or Microscan Walkaway-96SI (Siemens, Germany) automatic analyzer for bacterial identification and drug sensitivity analysis were used for bacterial identification, drug minimum inhibitory concentration (MIC) detection, and drug sensitivity analysis.

Culture medium: Mueller-Hinton agar (Becton, Dickinson and Company, United States) was used for the drug sensitivity test. *Streptococcus pneumoniae* and streptococcus groups using AGAR containing 5 ml/dl defibrated sheep blood (Autobio, China). Agara from Oxoid (Thermo Fisher Scientific, United Kingdom).

Reagents: Antibiotic paper and Epsilometer testing (Etest) of antibiotic resistance Etest strips (Thermo Fisher Scientific, United Kingdom).

Antibiotic susceptibility test disk: Levofloxacin (5 μg, Oxiod, United Kingdom), dalfopristin/quinupristin (15 μg, Oxiod, United Kingdom), Linezolid (30 μg, Oxiod, United Kingdom), Tigecycline (15 μg, Oxiod, United Kingdom), Moxifloxacin (5 μg, Oxiod, United Kingdom), Ciprofloxacin (5 μg, Oxiod, United Kingdom), Erythromycin (15 μg, Oxiod, United Kingdom), Furadantin (100 μg, Oxiod, United Kingdom), Gentamycin (10 μg, Oxiod, United Kingdom), Penicillin (10 μg, Oxiod, United Kingdom), Tetracycline (30 μg, Oxiod, United Kingdom), Clindamycin (2 μg, Oxiod, United Kingdom), Vancomycin (30 μg, Oxiod, United Kingdom), Cotrimoxazole (25 μg, Oxiod, United Kingdom), Piperacillin-tazobactam (40 μg, Oxiod, United Kingdom), Amikacin (30 μg, Oxiod, United Kingdom), Aztreonam (30 μg, Oxiod, United Kingdom), Ceftriaxone (30 μg, Oxiod, United Kingdom), Tobramycin (10 μg, Oxiod, United Kingdom), Cefepime (30 μg, Oxiod, United Kingdom), Gentamycin (10 μg, Oxiod, United Kingdom), Ertapenem (10 μg, Oxiod, United Kingdom).

Drug sensitivity test: The Disk diffusion antibiotic sensitivity testing method (also called Kirby- Bauer antibiotic testing or KB testing) or automatic drug sensitivity determination system was used. Kirby-Bauer disk diffusion method and Etest method were used to confirm the drug sensitivity results when in doubt. Screening of Methicillin-resistant *Staphylococcus aureus* (MRSA) by cefoxitin disk diffusion method. The Kirby-Bauer disk diffusion method was used for common Gram-negative bacteria (*E. coli*, *Klebsiella pneumoniae*, *Pseudomonas aeruginosa*, and *Acinetobacter baumannii*).

Re-check of drug sensitivity test: Rare drug resistance phenotypes were detected by Etest or other manual methods. For the detection of penicillin-insensitive *S. pneumoniae*, 1 ug/tablet of oxacillin was used to determine the inhibition zone of *S. pneumoniae*, and the penicillin Etest was further used to determine MIC values for those ≤19 mm. Detection of vancomycin-resistant enterococcus: For vancomycin-insensitive strains, MIC values were rechecked with the vancomycin-Etest. When CRE was detected by the Kirby-Bauer disk diffusion method, carbapenemases were detected by the Modified-Hodge test or Carba NP test. When the automated systems method and paper dispersion method were used for tigecycline, intermediate or drug-resistant bacteria appeared. We further tested the broth dilution method. Carbapenem-resistant strains were defined as resistant to any of the imipenem, meropenem, or ertapenem.

Setting of quality control strains: AST-GP6 test paper was used to detect *Staphylococcus aureus* ATCC29213 strain, AST-GP68 test paper was used to detect *S. pneumoniae* ATCC49619 strain, AST-GN13 test paper was used to detect *Escherichia coli* ATCC25922 strain, AST-GN09 test paper was used to detect *P. aeruginosa* ATCC27853 strain, and the quality control strain of *Enterococcus* was ATCC700327 (Purchased from Center of Clinical Laboratories of Ministry of Health, China).

The standard of antimicrobial susceptibility testing adhered to the M100 standards of the United States (Clinical and laboratory Standards Institute, CLSI), and used the M100-S22 to M100-S27 standards.

### Ethics Statement

All patient names and private data were kept anonymous and confidential. This study was approved by the Ethics Committee of West China Hospital of Sichuan University (2018-131) and complied with the requirements of the Helsinki Declaration. It was registered in the Chinese clinical trial center and received the registration number: ChiCTR1800017597.

### Statistical Analysis

The enumeration data were expressed as the number of cases (rate), and comparison between different groups was performed by the chi-square test or Fisher’s exact test. Binary logistic regression was used for performing multivariate analysis, and the “Enter” method was used for variable selection. Model calibration was tested with the Hosmer–Lemeshow test. We obtained the OR and 95% CI. *P* < 0.05 was considered statistically significant. IBM SPSS 21.0 software (SPSS Inc, Armonk, NY, United States) was used for data analysis, and GraphPad Prism 9.0.0 software or Excel (Microsoft) were used to draw statistical graphs.

## Results

### Demographics and Clinical Characteristics

From January 2012 to December 2017, a total of 9,900 patients with long bone fractures of the limbs were treated at West China Hospital. Among them, 535 patients (5.4%) had confirmed bone infections. There were 396 (74.0%) men and 139 (26.0%) women. The median age was 43 years (interquartile range [IQR], 31–52 years). In terms of age groups, the age group with the highest number of infections was 41–50 years, accounting for 157 cases (29.3%).

**FIGURE 2:**
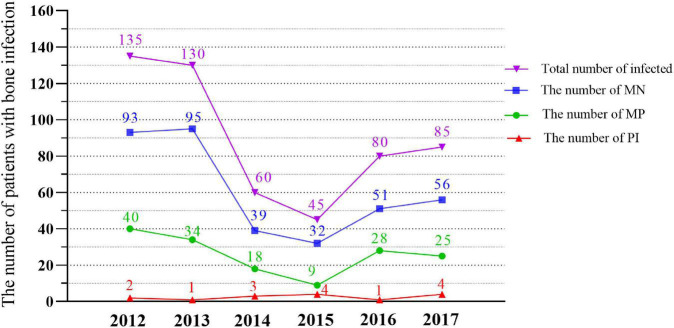
Quantitative trends of MN, MP, PI and the total Bacteria isolated in different years (2012–2017). MN, monomicr obial infection of negative bacteria; MP, monomicrobial infection of positive bacteria; PI, polymicrobial infection.

There were 424 (79.2%) open fractures, 37 closed fractures, and 74 unknown fracture types. The incidence of tibiofibula fractures (left 153 and right 145, a total of 298, 54.6%) was significantly higher than that at other sites. In terms of the causes of injury, traffic injuries was the most frequent cause with 282 cases (52.7%). In terms of comorbidities, 202 cases (37.8%) had no obvious comorbidities, while 121 cases (22.6%) had multiple fractures. There were 520 cases of monomicrobial infection (MI), including 154 cases of monomicrobial infection with Gram-positive bacteria (MP) and 366 cases of monomicrobial infection with Gram-negative bacteria (MN). The remaining 15 cases had polymicrobial infection (PI) (Supplementary Table 1). The overall trend of annual changes shows that the number of patients with bone infection is decreasing (Figure 2).

### Risk Factors for Fracture-Related Infection After Long Bone Fractures of the Extremities

#### Analysis of Risk Factors Associated With MP and MN Infections in the Population

A comparison of MN and MP infections showed that the proportion of MP infections in men was higher than that in women (*P* = 0.014). Among the three fracture types, open fracture type had the highest proportion of MP infections (*P* < 0.001). The proportion of MP infections in patients without comorbidities was higher than that in patients with comorbidities (*P* = 0.010). The proportion of MP infections in fractures due to traffic accidents was significantly higher than that from other causes (*P* = 0.001). Comparison of other factors between the groups showed no statistical difference (Supplementary Table 2).

**FIGURE 3:**
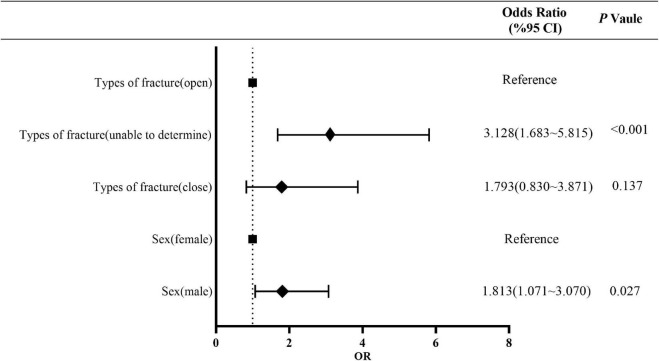
Multivariate risk factor analysis of MP population. MP, monomicrobial infection of positive bacteria.

The results of multifactorial risk factor analysis showed that sex, fracture type were the independent risk factors for MP infection. The risk of MP infection in men was 1.813 (95% confidence interval [CI], 1.071–3.070) times of that in women (*P* = 0.027). The risk of MP infection in patients with the fracture type “Unable to determine” was 3.128 (95% CI, 1.683–5.815) times of that in patients with an open fracture (*P* < 0.001), while there was no difference in the risk of MP between patients with close and open fractures (*P* = 0.137) (Figure 3 and Supplementary Table 3).

#### Risk Factors for Monomicrobial Infection and Polymicrobial Infection

**FIGURE 4:**
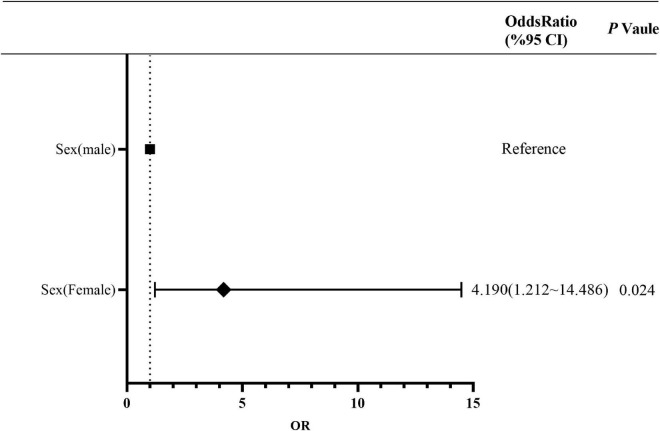
Multivariate risk factor analysis of PI population. PI, polymicrobial infection.

The proportion of PI in the 41–50 years age group was higher than that in other age groups (Supplementary Table 4). A comparison of the characteristics between MI and PI showed that the proportion of women was higher than that of men, and there was a statistically significant difference (*P* = 0.024). Multivariate risk factors analysis showed that sex was an independent risk factor for PI. The risk of PI in women was 4.190 (95% CI, 1.212–14.486) times of that in men (Figure 4 and Supplementary Table 5). In addition, there was no statistically significant difference among other characteristics.

#### Bacterial Statistics

**FIGURE 5:**
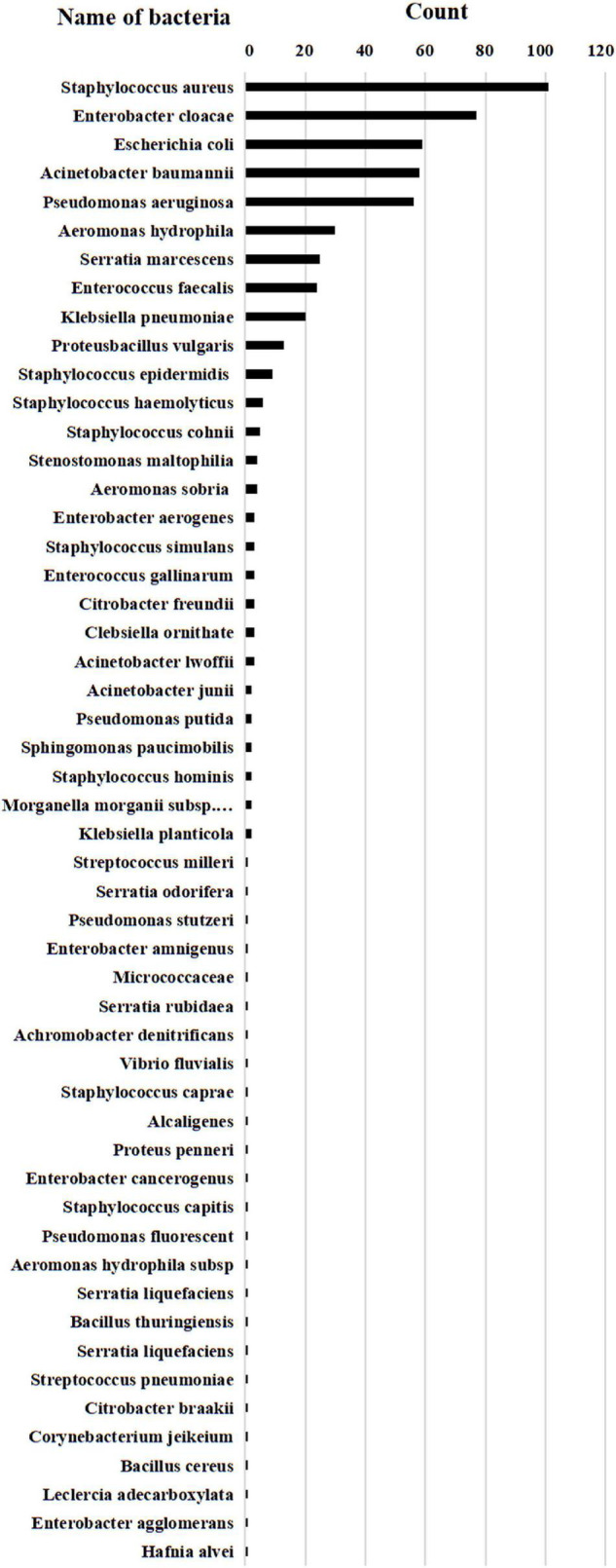
Bacterial statistics. A total of 546 strains of 52 species of bacteria. A total of 101 strains of Staphylococcus aureus (including MRSA 48 strains, MSSA 53 strains), 77 strains of Enterobacter cloacae (including Enterobacter cloacae 72 strains, CRECL 5 strains), 59 strains of Escherichia coli (including Escherichia coli 27 strains and ESBL Escherichia coli 32 strains), 58 strains of Acinetobacter baumannii (including Acinetobacter baumannii 55 strains and CRAB 3 strains), 56 strains of Pseudomonas aeruginosa (including Pseudomonas aeruginosa 47 strains and MDR Pseudomonas aerogenosa 9 strains), 20 strains of Klebsiella pneumoniae (including Klebsiella pneumoniae 12 strains and ESBL Klebsiella pneumoniae 8 strains).

We isolated a total of 546 strains of 52 species of bacteria. *S. aureus* (101 strains, 18.4%) accounted for the majority, including 48 strains of MRSA and 53 strains of methicillin-sensitive *S. aureus* (MSSA). Followed in descending order by *Enterobacter cloacae* (77 strains, 14.1%), *E. coli* (10.8%), *A. baumannii* (58 strains, 10.6%), and *P. aeruginosa* (56 strains, 10.2%). In addition, a total of 105 multidrug-resistant (MDR) bacterial infections were found in our study, including 48 strains of MRSA (8.8%), three strains of carbapenem-resistant *A. baumannii* (CRAB) (0.5%), nine strains of MDR *P. aeruginosa* (1.6%), eight strains of extended-spectrum-β-lactamase (ESBL)-producing *K. pneumoniae* (1.4%), 32 strains of ESBL *E. coli* (5.8%), and five strains of carbapenem-resistant *Enterobacter cloacae* (CRECL) (0.9%) (Figure 5 and Supplementary Table 6).

#### Analysis of Bacterial Resistance

**FIGURE 6:**
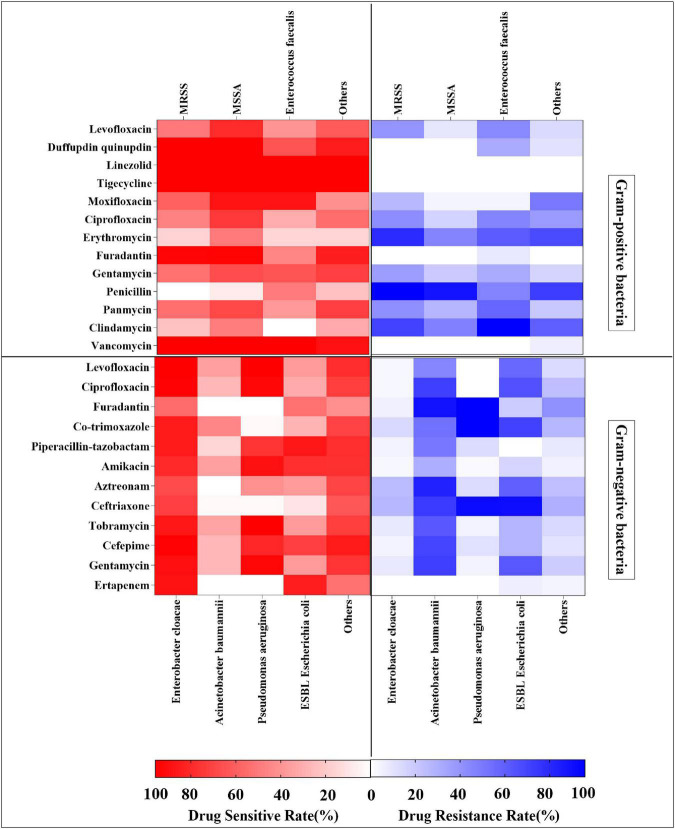
Heat map showing drug resistance rates and sensitivity rates analysis of Gram-positive bacteria and Gram-negative bacteria.

Methicillin-resistant *Staphylococcus aureus* showed high rates of drug resistance to penicillin (100%), erythromycin (82.9%), and clindamycin (73.9%), but was sensitive to tigecycline (100%), linezolid (100%), vancomycin (100%), dalfopristin/quinupristin (100%), furadantin (97.9%), and moxifloxacin (61.7%). MSSA had high drug resistance rates to penicillin (92.4%) and clindamycin (49.0%), but was sensitive to tigecycline (100%), linezolid (100%), vancomycin (100%), dalfopristin/quinupristin (100%), furadantin (98.1%), and moxifloxacin (92.1%). *Enterococcus faecalis* was resistant to clindamycin (100%) and tetracycline (60.0%), but sensitive to vancomycin (100%), tigecycline (100%), linezolid (100%), and moxifloxacin (92.1%). Other Gram-positive bacteria were 100% sensitive to linezolid and tigecycline, and 93.5% to vancomycin, whereas they were more than 70% resistant to penicillin and erythromycin (Figure 6 and Supplementary Table 7). Among Gram-negative bacteria, the drug resistance rates of *A. baumannii* to furadantin, aztreonam, ceftriaxone, gentamicin, and ciprofloxacin all exceeded 70%. The resistance rates of ESBL *E. coli* to ceftriaxone and cotrimoxazole were 90.6 and 71.9%, respectively, while the sensitivity rates to ertapenem, piperacillin-tazobactam, amikacin, and cefepime were 84.4, 87.5, 78.1, and 71.9%, respectively. The resistance rates of *P. aeruginosa* to furadantin, co-trimoxazole, and ceftriaxone were higher than 90.0%, and the sensitivity rates to levofloxacin, amikacin, tobramycin, and gentamycin were more than 89%. *E. cloacae* demonstrated more than 80% sensitivity to levofloxacin, ciprofloxacin, amikacin, and cefepime. The remaining Gram-negative bacteria were more than 70% sensitive to levofloxacin, piperacillin-tazobactam, amikacin, and cefepime. In addition, the drug resistance rate for furadantin was 41.4%, while the drug resistance rates for piperacillin-tazobactam, amikacin, and ertapenem were 10% or less (Figure 6 and Supplementary Table 8).

## Discussion

This is the first retrospective study conducted at West China Hospital in western China to describe the characteristics, site, fracture type, pathogen distribution, drug resistance, and risk factors for FRI following long bone fractures of the extremities. In our study, middle-aged and young men were the main high-risk population (396,74.0%) for FRI in the long bones of the extremities, with a significantly higher incidence among men compared to women, which was similar to the results of previous studies (Jiang et al., 2015; Peng et al., 2017). In terms of the causes, traffic accident injuries (282, 52.7%)were the main cause of FRI, indicating that road traffic safety is a very serious issue (Zhou et al., 2014; Ning et al., 2016). At the same time, we observed that injuries from high fall and hard object also belonged to the category of high energy injury, which is also an important cause of bone infection. The incidence of tibiofibula infection was 55.7%, which was consistent with the results of previously studies (Ma et al., 2018; Bezstarosti et al., 2019; Zhang et al., 2019). This may be because of the less soft tissue coverage and easy inactivation of the lower limbs, making them more prone to open fractures of the tibiofibula.

Previous studies have shown that *S. aureus* is a common bacterial cause of FRI of the long bones of the extremities (Masters et al., 2019; Saeed et al., 2019). Our study results revealed that *S. aureus* had the highest number of strains (101, 18.4%), with MRSA accounted for 8.7% of the total strains and 47.5% of *S. aureus*, which was lower than the 11.4% reported by Dudareva et al. (2019) and 34.7% reported by Jorge et al. (2018). Then came *E. cloacae*, *A. baumannii*, and *P. aeruginosa*, whose bacterial spectrum was similar to that reported in previous studies (Wang et al., 2017; Ma et al., 2018; Kuehl et al., 2019). The proportion of PI in our study was 2.8%, which was significantly lower than the 20–37.8% of traumatic PI reported by Jiang et al. (2015) and Jorge et al. (2018). At the same time, we noted that the total number of infections showed a downward trend with the passage of time, which may be related to our trauma treatment policy and the improvement in medical standards. No fungus-induced FRI was observed in our study, which occurs most often in immunosuppressed populations and in those receiving long-term non-standard broad-spectrum antibiotic therapy (De Meo et al., 2021).

In multivariate analysis, sex, fracture type were the independent risk factors for MP. More men than women had PI. Consistent with the results of previous studies (Ma et al., 2018), the prevalence of multiple infections was higher in the 41–50 years age group than in other age groups, which may be related to the high incidence of fractures in the 41–50 years age group, as this is the main labor force in developing countries. However, this is different from the age group reported by Walter et al. (2021) (patients aged 70–79 years old were the largest cohort, accounting for 20.7% cases), which may be related to the population decline and aging in developed European and American countries. The overall physical condition of the host has been regarded as an important factor for the prognosis of osteomyelitis (Parkkinen et al., 2016). In our study, multiple fractures, shock, and vascular damage at the site were risk factors for infection in terms of the comorbidities, which was different from the proportion of patients with diabetes reported by Kremers et al. (2015).

In recent years, secondary MDR microbial infections have become an increasingly prominent problem in FRI of the limb long bones (Kavanagh et al., 2018). The use of antibiotics and increase in bacterial resistance have become a global public health problem. In our study, MRSA, MSSA, and *E. faecalis* were more sensitive to tigecycline, linezolid, vancomycin, and moxifloxacin, but more resistant to penicillin and clindamycin. It is gratifying that vancomycin-resistant *S. aureus* (VRSA) was not isolated in our study population despite the increasing prevalence of VRSA (Conly and Johnston, 2002; Rehman et al., 2020). β-lactamase inhibitors are often combined with β -lactamase antibiotics to exert a therapeutic effect on Gram-negative bacteria (Probst-Kepper and Geginat, 2018; Ibrahim et al., 2019). Our results showed that *A. baumannii*, *P. aeruginosa*, and *E. cloacae* were resistant to piperacillin-tazobactam, but were more sensitive to amikacin. In addition, we found that two ESBL *E. coli* strains and five CRECL strains were resistant to ertapenem. Inappropriate empiric antibiotic use may be one of the risk factors for MDR (Bassetti and Garau, 2021). This may bring new challenges in the treatment of FRI in the future.

There are several limitations of this study. First, the study encompassed a 6-year time span during which trauma care and prevention protocols underwent a change. Secondly, since this was a single-center retrospective study, the patients included in the study are not fully representative of the situation in other regions and may be affected by a confusion bias. Thirdly, due to the limited sample size, we could not provide sufficient statistical data for an in-depth analysis of the infection-related clinical factors. Moreover, we did not perform genome sequencing of the isolated bacteria. In conclusion, the results should be interpreted with caution after considering the limitations of the study. Nevertheless, this study facilitates comparisons with past situations in the same country and elsewhere, although further efforts are needed to address the limitations and obtain precise results. Considering the complexity and diversity of risk factors for infection in the long bones of the extremities, medical staff should strengthen training, improve the treatment capacity, and ensure strict implementation of aseptic techniques in addition to promoting the rational use of antibiotics. Management of FRI in the future will be challenging and requires an in-depth exploration.

## Conclusion

This study clarified the infection rates, changes in the bacterial spectrum, drug resistance characteristics, and risk factors of FRI of the long bones of the extremities in the largest trauma center in southwest China. Gender, fracture type, and falling injury were independent risk factors for MI of MP, while the female was an independent risk factor for PI.

## Data Availability Statement

The datasets presented in this article are not readily available because all data are available in the article and attachments. Requests to access the datasets should be directed to LL, liuinsistence@163.com.

## Ethics Statement

This study was approved by the West China Hospital, Sichuan University Ethics Committee, Sichuan University (2018-131).

## Author Contributions

ZZ and PL collected data, performed the statistical analysis, and wrote the manuscript. WW, SW, BL, and JL performed data analysis. BY, ML, QL, and HY did data collection. ZZ, ZH, and LL was the director of this study. All authors contributed to the article and approved the submitted version.

## Conflict of Interest

The authors declare that the research was conducted in the absence of any commercial or financial relationships that could be construed as a potential conflict of interest.

## Publisher’s Note

All claims expressed in this article are solely those of the authors and do not necessarily represent those of their affiliated organizations, or those of the publisher, the editors and the reviewers. Any product that may be evaluated in this article, or claim that may be made by its manufacturer, is not guaranteed or endorsed by the publisher.
