# Supplementary material for: Epidemiology and Drug Resistance of Fracture-Related Infection of the Long Bones of the Extremities: A Retrospective Study at the Largest Trauma Center in Southwest China
Source: Front Microbiol. 2022 Jul 12;13:923735. doi: 10.3389/fmicb.2022.923735 (PMC9315197; doi:10.3389/fmicb.2022.923735)
Supplement: Supplementary file 1 [file Table_1.docx]

**Supplementary**

Table 1. Characteristic analysis of patients with FRI.

|  |  | MP (n=154) | MN (n=366) | PI (n=15) | χ^2^ | *P* value |
| --- | --- | --- | --- | --- | --- | --- |
| Nationality |  |  |  |  | 3.660 | 0.454 |
|  | Han Nationality | 145(94.2%) | 345(94.3%) | 14(93.3%) |  |  |
|  | Tibetan nationality | 5(3.2%) | 18(4.9%) | 1(6.7%) |  |  |
|  | Yi nationality | 4(2.6%) | 3(0.8%) | 0 |  |  |
| Sex |  |  |  |  | 9.336 | 0.009 |
|  | Male | 126(81.8%) | 262(71.6%) | 8(53.3%) |  |  |
|  | Female | 28(18.2%) | 104(28.4%) | 7(46.7%) |  |  |
| Age |  |  |  |  | 15.114 | 0.128 |
|  | ≤ 20 years | 13 (8.4%) | 29(7.9%) | 0 |  |  |
|  | 21－30 years | 22(14.3%) | 59(16.1%) | 3(20.0%) |  |  |
|  | 31－40 years | 29(18.8%) | 75(20.5%) | 0 |  |  |
|  | 41－50 years | 50(32.5%) | 97(26.5%) | 10(66.7%) |  |  |
|  | 51－60 years | 22(14.3%) | 62(16.9%) | 1(6.7%) |  |  |
|  | ≥ 60years | 18(11.7%) | 44(12.0%) | 1(6.7%) |  |  |
| Fracture types |  |  |  |  | 39.902 | <0.001 |
|  | Open fracture | 97(63.0%) | 312(85.2%) | 15(100.0%) |  |  |
|  | Close fracture | 15(9.7%) | 22(6.0%) | 0 |  |  |
|  | Unable to determine | 42(27.3%) | 32(8.7%) | 0 |  |  |
| Fracture sites |  |  |  |  | 11.454 | 0.650 |
|  | Tibiofibular (L) | 39(25.3%) | 110(30.1%) | 4(26.7%) |  |  |
|  | Femur (L) | 26(16.9%) | 55(15.0%) | 3(20.0%) |  |  |
|  | Humerus (L) | 4(2.6%) | 9(2.5%) | 1(6.7%) |  |  |
|  | Ulna and radius (L) | 5(3.2%) | 14(3.8%) | 1(6.7%) |  |  |
|  | Tibiofibular (R) | 43(27.9%) | 97(26.5%) | 5(33.3%) |  |  |
|  | Femur (R) | 20(13.0%) | 47(12.8%) | 0 |  |  |
|  | Humerus (R) | 4(2.6%) | 19(5.2%) | 1(6.7%) |  |  |
|  | Ulna and radius (R) | 13(8.4%) | 15(4.1%) | 0 |  |  |
| Cause of injury |  |  |  |  | 32.429 | 0.003 |
|  | Hard object injury | 9(5.8%) | 45(12.3%) | 2(13.3%) |  |  |
|  | Falling injury | 36(23.4%) | 48(13.1%) | 1(6.7%) |  |  |
|  | Traffic accident | 73(47.4%) | 203(55.5%) | 6(40.0%) |  |  |
|  | Destructive injury | 6(3.9%) | 27(7.4%) | 3(20.0%) |  |  |
|  | Penetrating injury of sharp force | 2(1.3%) | 8(2.2%) | 1(6.7%) |  |  |
|  | Crush injury | 15(9.7%) | 24(6.6%) | 2(13.3%) |  |  |
|  | Other physical high-energy damage (Such as blast injuries ) | 2(1.3%) | 1(0.3%) | 0 |  |  |
|  | Others | 11(7.1%) | 10(2.7%) | 0 |  |  |
| Comorbidity |  |  |  |  | 28.126 | 0.031 |
|  | None | 75(48.7%) | 124(33.9%) | 3(20.0%) |  |  |
|  | Diabetes | 4(2.6%) | 8(2.2%) | 0 |  |  |
|  | Anemia | 4(2.6%) | 8(2.2%) | 1(6.7%) |  |  |
|  | Chronic medical diseases | 6(3.9%) | 6(1.6%) | 0 |  |  |
|  | Shock | 12(7.8%) | 70(19.1%) | 3(20.0%) |  |  |
|  | Multiple fracture | 27(17.5%) | 88(24.0%) | 6(40.0%) |  |  |
|  | Vascular injury at the fracture site | 12(7.8%) | 35(9.6%) | 2(13.3%) |  |  |
|  | Nerve injury at the fracture site | 6(3.9%) | 8(2.2%) | 0 |  |  |
|  | With internal organ injury | 8(5.2%) | 19(5.2%) | 0 |  |  |

Abbreviations: FRI, Fracture-related infection. MP, monomicrobial infection of positive bacteria; MN, monomicrobial infection of negative bacteria; PI, polymicrobial infection; R, right; L, left.

Table 2. Comparison of patient characteristics between MN and MP.

|  |  | MN (n=366) | MP (n=154) | χ^2^ | *P*value |
| --- | --- | --- | --- | --- | --- |
| Nationality |  |  |  |  | 0.219^#^ |
|  | Han Nationality | 345(94.3%) | 145(94.2%) |  |  |
|  | Tibetan nationality | 18(4.9%) | 5(3.2%) |  |  |
|  | Yi nationality | 3(0.8%) | 4(2.6%) |  |  |
| Sex |  |  |  | 5.993 | 0.014 |
|  | Male | 262(71.6%) | 126(81.8%) |  |  |
|  | Female | 104(28.4%) | 28(18.2%) |  |  |
| Fracture types |  |  |  | 35.098 | <0.001 |
|  | Open fracture | 312(85.2%) | 97(63.0%) |  |  |
|  | Close fracture | 22(6.0%) | 15(9.7%) |  |  |
|  | Unable to determine | 32(8.7%) | 42(27.3%) |  |  |
| Fracture sites |  |  |  |  | 0.462^#^ |
|  | Tibiofibular (L) | 110(30.1%) | 39(25.7%) |  |  |
|  | Femur (L) | 55(15.0%) | 27(17.5%) |  |  |
|  | Humerus (L) | 9(2.5%) | 4(2.6%%) |  |  |
|  | Ulna and radius (L) | 14(3.8%) | 5(3.2%) |  |  |
|  | Tibiofibular (R) | 97(26.5%) | 42(27.3%) |  |  |
|  | Femur (R) | 47(12.8%) | 20(13.0%) |  |  |
|  | Humerus (R) | 19(5.2%) | 4(2.6%) |  |  |
|  | Ulna and radius (R) | 15(4.1%) | 13(8.4%) |  |  |
| Age |  |  |  |  | 0.813^#^ |
|  | ≤ 20 years | 29(7.9%) | 13(8.4%) |  |  |
|  | 21－30 years | 59(16.1%) | 22(14.3%) |  |  |
|  | 31－40 years | 75(20.5%) | 29(18.8%) |  |  |
|  | 41－50 years | 97(26.5%) | 50(32.5%) |  |  |
|  | 51－60 years | 62(16.9%) | 22(14.3%) |  |  |
|  | ≥ 60years | 44(12.0%) | 18(11.7%) |  |  |
| Comorbidity |  |  |  |  | 0.010^#^ |
|  | None | 124(33.9%) | 75(48.7%) |  |  |
|  | Diabetes | 8(2.2%) | 4(2.6%) |  |  |
|  | Anemia | 8(2.2%) | 4(2.6%) |  |  |
|  | Chronic medical diseases | 6(1.6%) | 6(3.9%) |  |  |
|  | Shock | 70(19.1%) | 12(7.8%) |  |  |
|  | Multiple fracture | 88(24.0%) | 27(17.5%) |  |  |
|  | Vascular injury at the fracture site | 35(9.6%) | 12(7.8%) |  |  |
|  | Nerve injury at the fracture site | 8(2.2%) | 6(3.9%) |  |  |
|  | With internal organ injury | 19(5.2%) | 8(5.2%) |  |  |
| Cause of injury |  |  |  |  | 0.001^#^ |
|  | Hard object injury | 45(12.3%) | 9(5.8%) |  |  |
|  | Falling injury | 48(13.1%) | 36(23.4%) |  |  |
|  | Traffic accident | 203(55.5%) | 73(47.4%) |  |  |
|  | Destructive injury | 27(7.4%) | 6(3.9%) |  |  |
|  | Penetrating injury of sharp force | 8(2.2%) | 2(1.3%) |  |  |
|  | Crush injury | 24(6.6%) | 15(9.7%) |  |  |
|  | Other physical high-energy damage (Such as blast injuries ) | 1(0.3%) | 2(1.3%) |  |  |
|  | Others | 10(2.7%) | 11(7.1%) |  |  |

Abbreviations: MP: monomicrobial infection of positive bacteria; MN: monomicrobial infection of negative bacteria.

^#^ Fisher's exact test.

Table 3. Results of multivariate risk factors analysis in MP and MN populations.

|  |  | β | S.E. | OR（95%CI） | Wald value | *P* value |
| --- | --- | --- | --- | --- | --- | --- |
| Sex | Female |  |  | 1.00 |  |  |
|  | Male | 0.595 | 0.269 | 1.813(1.071~3.070) | 4.904 | 0.027 |
| Types of Fracture | Open fracture |  |  | 1.00 |  |  |
|  | Close fracture | 0.584 | 0.393 | 1.793(0.830~3.871) | 2.210 | 0.137 |
|  | Unable to determine | 1.141 | 0.316 | 3.128(1.683~5.815) | 13.005 | <0.001 |

Abbreviations: OR, odds ratio.

Table 4. Comparison of patient characteristics between MI and PI.

|  |  | MI (n=520) | PI (n=15) | χ^2^ | *P* value |
| --- | --- | --- | --- | --- | --- |
| Nationality |  |  |  |  | 0.597^#^ |
|  | Han Nationality | 490(94.2%) | 14(93.3%) |  |  |
|  | Tibetan nationality | 23(4.4%) | 1(6.7) |  |  |
|  | Yi nationality | 7(1.3%) | 0 |  |  |
| Sex |  |  |  | 2.416 | 0.120 |
|  | Male | 388(74.6%) | 8(53.3%) |  |  |
|  | Female | 132(25.4%) | 7(46.7%) |  |  |
| Fracture types |  |  |  | 4.040 | 0.133 |
|  | Open fracture | 409(78.7%) | 15(100.0%) |  |  |
|  | Close fracture | 37(7.1%) | 0 |  |  |
|  | Unable to determine | 74(14.2%) | 0 |  |  |
| Fracture sites |  |  |  |  | 0.451^#^ |
|  | Tibiofibular (L) | 149(28.7%) | 4(26.7%) |  |  |
|  | Femur (L) | 81(15.6%) | 3(20.0%) |  |  |
|  | Humerus (L) | 13(2.5%) | 1(6.7%) |  |  |
|  | Ulna and radius (L) | 19(3.7%) | 1(6.7%) |  |  |
|  | Tibiofibular (R) | 140(26.9%) | 5(33.3%) |  |  |
|  | Femur (R) | 67(12.9%) | 0(0.0%) |  |  |
|  | Humerus (R) | 23(4.4%) | 1(6.7%) |  |  |
|  | Ulna and radius (R) | 28(5.4%) | 0 |  |  |
| Age |  |  |  |  | 0.029^#^ |
|  | ≤ 20 years | 42(8.1%) | 0 |  |  |
|  | 21－30 years | 81(15.6%) | 3(20.0%) |  |  |
|  | 31－40 years | 104(20.0%) | 0 |  |  |
|  | 41－50 years | 147(28.3%) | 10(66.7%) |  |  |
|  | 51－60 years | 84(16.2%) | 1(6.7%) |  |  |
|  | ≥ 60years | 62(11.9%) | 1(6.7%) |  |  |
| Comorbidity |  |  |  |  | 0.488^#^ |
|  | None | 199(38.3%) | 3(20.0%) |  |  |
|  | Diabetes | 12(2.3%) | 0 |  |  |
|  | Anemia | 12(2.3%) | 1(6.7%) |  |  |
|  | Chronic medical diseases | 12(2.3%) | 0 |  |  |
|  | Shock | 82(15.8%) | 3(20.0%) |  |  |
|  | Multiple fracture | 115(22.1%) | 6(40.0%) |  |  |
|  | Vascular injury at the fracture site | 47(9.0%) | 2(13.3%) |  |  |
|  | Nerve injury at the fracture site | 14(2.7%) | 0 |  |  |
|  | With internal organ injury | 27(5.2%) | 0 |  |  |
| Cause of injury |  |  |  |  | 0.176^#^ |
|  | Hard object injury | 54(10.4%) | 2(13.3%) |  |  |
|  | Falling injury | 84(16.2%) | 1(6.7%) |  |  |
|  | Traffic accident | 276(53.1%) | 6(40.0%) |  |  |
|  | Destructive injury | 33(6.3%) | 3(20.0%) |  |  |
|  | Penetrating injury of sharp force | 10(1.9%) | 1(6.7%) |  |  |
|  | Crush injury | 39(7.5%) | 2(13.3%) |  |  |
|  | Other physical high-energy damage (Such as blast injuries ) | 3(0.6%) | 0 |  |  |
|  | Others | 21(4.0%) | 0 |  |  |

^#^ Fisher's exact test.

Table 5. Result of multivariate risk factors analysis in MI and PI populations.

|  |  | β | S.E. | OR（95%CI） | Wald value | *P* value |
| --- | --- | --- | --- | --- | --- | --- |
| Gender | Male |  |  | 1 |  |  |
|  | Female | 1.433 | 0.633 | 4.190(1.212~14.486) | 5.126 | 0.024 |

Abbreviations: OR, odds ratio.

Table 6. The species of bacteria and the number of different bacteria. We isolated a total of 546 strains of 52 species of bacteria. A total 101 strains of Staphylococcus aureus (including MRSA 48 strains, MSSA 53 strains), 77 strains of Enterobacter cloacae (including Enterobacter cloacae 72 strains, CRECL 5 strains), 59 strains of Escherichia coli (including Escherichia coli 27 strains and ESBL Escherichia coli 32 strains), 58 strains of Acinetobacter baumannii (including Acinetobacter baumannii 55 strains and CRAB 3 strains), 56 strains of Pseudomonas aeruginosa (including Pseudomonas aeruginosa 47 strains and MDR Pseudomonas aerogenosa 9 strains), 20 strains of Klebsiella pneumoniae (including Klebsiella pneumoniae 12 strains and ESBL Klebsiella pneumoniae 8 strains).

| **Name of bacteria** | **Count** |
| --- | --- |
| Staphylococcus aureus | 101 |
| Enterobacter cloacae | 77 |
| Escherichia coli | 59 |
| Acinetobacter baumannii | 58 |
| Pseudomonas aeruginosa | 56 |
| Aeromonas hydrophila | 30 |
| Serratia marcescens | 25 |
| Enterococcus faecalis | 24 |
| Klebsiella pneumoniae | 20 |
| Proteusbacillus vulgaris | 13 |
| Staphylococcus epidermidis | 9 |
| Staphylococcus haemolyticus | 6 |
| [Staphylococcus cohnii](../../../../../../../%E5%BC%A0%E6%AD%A3%E4%B8%9C/AppData/Local/youdao/dict/Application/8.9.9.0/resultui/html/index.html) | 5 |
| Stenostomonas maltophilia | 4 |
| Aeromonas sobria | 4 |
| Enterobacter aerogenes | 3 |
| Staphylococcus simulans | 3 |
| Enterococcus gallinarum | 3 |
| Citrobacter freundii | 3 |
| Clebsiella ornithate | 3 |
| Acinetobacter lwoffii | 3 |
| Acinetobacter junii | 2 |
| Pseudomonas putida | 2 |
| Sphingomonas paucimobilis | 2 |
| Staphylococcus hominis | 2 |
| Morganella morganii subsp. Morganii | 2 |
| Klebsiella planticola | 2 |
| Streptococcus milleri | 1 |
| Serratia odorifera | 1 |
| Pseudomonas stutzeri | 1 |
| Enterobacter amnigenus | 1 |
| Micrococcaceae | 1 |
| Serratia rubidaea | 1 |
| Achromobacter denitrificans | 1 |
| Vibrio fluvialis | 1 |
| Staphylococcus caprae | 1 |
| Alcaligenes | 1 |
| Proteus penneri | 1 |
| Enterobacter cancerogenus | 1 |
| Staphylococcus capitis | 1 |
| Pseudomonas fluorescent | 1 |
| Aeromonas hydrophila subsp | 1 |
| Serratia liquefaciens | 1 |
| Bacillus thuringiensis | 1 |
| Serratia liquefaciens | 1 |
| Streptococcus pneumoniae | 1 |
| Citrobacter braakii | 1 |
| Corynebacterium jeikeium | 1 |
| Bacillus cereus | 1 |
| Leclercia adecarboxylata | 1 |
| Enterobacter agglomerans | 1 |
| Hafnia alvei | 1 |

Abbreviations: MRSA, Methicillin-resistant Staphylococcus aureus; MSSA, Methicillin-sensitive Staphylococcus aureus; CRECL, Carbapenem-resistant Enterobacter cloacae; ESBL, Extended-spectrum-β-lactamase; MDR, Multidrug-resistant; CRAB, Carbapenem-resistant Acinetobacter baumannii; MDR: Multidrug-resistant.

Table 7. Analysis of drug resistance rates and sensitivity rates of Gram-positive bacteria.

| Drug | MRSA | | MSSA | | Enterococcus faecalis | | Others | |
| --- | --- | --- | --- | --- | --- | --- | --- | --- |
|  | Sensitivity(%) | Resistance(%) | Sensitivity(%) | Resistance(%) | Sensitivity(%) | Resistance(%) | Sensitivity(%) | Resistance(%) |
| Levofloxacin | 52.17 | 41.30 | 83.02 | 9.43 | 41.67 | 45.83 | 64.29 | 14.29 |
| dalfopristin/ quinupristin | 100.00 | 0.00 | 100.00 | 0.00 | 66.67 | 33.33 | 88.89 | 11.11 |
| Linezolid | 100.00 | 0.00 | 100.00 | 0.00 | 100.00 | 0.00 | 100.00 | 0.00 |
| Tigecycline | 100.00 | 0.00 | 100.00 | 0.00 | 100.00 | 0.00 | 100.00 | 0.00 |
| Moxifloxacin | 61.70 | 27.66 | 92.16 | 3.92 | 92.16 | 3.92 | 43.48 | 52.17 |
| Ciprofloxacin | 48.94 | 44.68 | 77.36 | 16.98 | 32.00 | 48.00 | 57.14 | 39.29 |
| Erythromycin | 17.02 | 82.98 | 51.92 | 48.08 | 16.00 | 64.00 | 16.13 | 70.97 |
| Furadantin | 97.96 | 0.00 | 98.11 | 0.00 | 47.83 | 8.70 | 87.50 | 0.00 |
| Gentamycin | 55.32 | 38.30 | 69.81 | 20.75 | 66.67 | 33.33 | 75.00 | 16.67 |
| Penicillin | 0.00 | 100.00 | 7.55 | 92.45 | 52.00 | 48.00 | 24.14 | 75.86 |
| Tetracycline | 56.25 | 43.75 | 71.70 | 28.30 | 40.00 | 60.00 | 75.00 | 21.43 |
| Clindamycin | 23.91 | 73.91 | 50.94 | 49.06 | 0.00 | 100.00 | 33.33 | 62.50 |
| Vancomycin | 100.00 | 0.00 | 100.00 | 0.00 | 100.00 | 0.00 | 93.55 | 6.45 |

Abbreviations: MSSA, Methicillin-sensitive Staphylococcus aureus; MRSA, Methicillin-resistant Staphylococcus aureus.

Table 8. Analysis of drug resistance rates and sensitivity rates of Gram-negative bacteria.

| Drug | Acinetobacter baumannii | | ESBL Escherichia coli | | Pseudomonas aeruginosa | | Enterobacter cloacae | | Others | |
| --- | --- | --- | --- | --- | --- | --- | --- | --- | --- | --- |
|  | Sensitivity(%) | Resistance(%) | Sensitivity(%) | Resistance(%) | Sensitivity(%) | Resistance(%) | Sensitivity(%) | Resistance(%) | Sensitivity(%) | Resistance(%) |
| Levofloxacin | 35.8 | 45.3 | 37.5 | 56.3 | 95.7 | 0.00 | 95.8 | 2.8 | 78.7 | 13.2 |
| Ciprofloxacin | 26.4 | 71.7 | 31.3 | 65. 6 | 93.5 | 0.00 | 94.4 | 2.8 | 72.4 | 24.1 |
| Furadantin | 0.00 | 88.7 | 53.1 | 18.8 | 0.00 | 95.7 | 54.9 | 5.6 | 42.0 | 41.4 |
| Cotrimoxazole | 45.30 | 52.80 | 28.1 | 71. 9 | 2.2 | 95.7 | 85.9 | 14.1 | 70.70 | 27.00 |
| Piperacillin-tazobactam | 15.1 | 50.9 | 87.5 | 0.00 | 76.1 | 13.0 | 85.9 | 4.2 | 78.2 | 8.6 |
| Amikacin | 35.8 | 30.2 | 78.1 | 15.6 | 89.1 | 2.2 | 80.3 | 2.8 | 77.6 | 5.2 |
| Aztreonam | 0.00 | 83.0 | 37.5 | 59.4 | 41.3 | 13.0 | 67.6 | 25.4 | 70.1 | 23.6 |
| Ceftriaxone | 1.9 | 73.6 | 9.4 | 90.6 | 2.2 | 91.3 | 71.8 | 26.8 | 63.2 | 29.9 |
| Tobramycin | 34.0 | 62.3 | 37.5 | 28.1 | 95.7 | 4.3 | 87.3 | 8.5 | 72.4 | 14.4 |
| Cefepime | 26.4 | 69.8 | 71.9 | 28.1 | 80.4 | 10.9 | 94.4 | 4.2 | 85.6 | 10.3 |
| Gentamycin | 26.4 | 71.7 | 37.5 | 62.5 | 93.5 | 4.3 | 90.1 | 8.5 | 75.9 | 19.0 |
| Ertapenem | - | - | 84.4 | 6.3 | - | - | 88.7 | 0.0 | 52.9 | 4.0 |

Abbreviations: ESBL, Extended-spectrum-β-lactamase.
